# Efficient Protoplast Regeneration Protocol and CRISPR/Cas9-Mediated Editing of Glucosinolate Transporter (*GTR*) Genes in Rapeseed (*Brassica napus* L.)

**DOI:** 10.3389/fpls.2021.680859

**Published:** 2021-07-07

**Authors:** Xueyuan Li, Sjur Sandgrind, Oliver Moss, Rui Guan, Emelie Ivarson, Eu Sheng Wang, Selvaraju Kanagarajan, Li-Hua Zhu

**Affiliations:** Department of Plant Breeding, Swedish University of Agricultural Sciences, Lomma, Sweden

**Keywords:** *Brassica napus*, CRISPR/Cas9, gene editing, glucosinolate transporter, *GTR* gene, protoplast regeneration

## Abstract

Difficulty in protoplast regeneration is a major obstacle to apply the CRISPR/Cas9 gene editing technique effectively in research and breeding of rapeseed (*Brassica napus* L.). The present study describes for the first time a rapid and efficient protocol for the isolation, regeneration and transfection of protoplasts of rapeseed cv. Kumily, and its application in gene editing. Protoplasts isolated from leaves of 3–4 weeks old were cultured in MI and MII liquid media for cell wall formation and cell division, followed by subculture on shoot induction medium and shoot regeneration medium for shoot production. Different basal media, types and combinations of plant growth regulators, and protoplast culture duration on each type of media were investigated in relation to protoplast regeneration. The results showed that relatively high concentrations of NAA (0.5 mg l^−1^) and 2,4-D (0.5 mg l^−1^) in the MI medium were essential for protoplasts to form cell walls and maintain cell divisions, and thereafter auxin should be reduced for callus formation and shoot induction. For shoot regeneration, relatively high concentrations of cytokinin were required, and among all the combinations tested, 2.2 mg l^−1^ TDZ in combination with auxin 0.5 mg l^−1^ NAA gave the best result with up to 45% shoot regeneration. Our results also showed the duration of protoplast culture on different media was critical, as longer culture durations would significantly reduce the shoot regeneration frequency. In addition, we have optimized the transfection protocol for rapeseed. Using this optimized protocol, we have successfully edited the *BnGTR* genes controlling glucosinolate transport in rapeseed with a high mutation frequency.

## Introduction

The CRISPR/Cas9 technology has now become a prevailing tool for plant genome editing owing to its high precision, efficiency and simplicity in use (Arora and Narula, 2017). Apart from its powerful role in functional genomics analysis, it has also revolutionized the strategy for crop breeding and improvement. So far the CRISPR/Cas9 system has been successfully applied to edit genes in a number of plant species, such as *Arabidopsis thaliana* (Jiang et al., 2013), *Nicotiana tabacum* (Nekrasov et al., 2013), rice (Shan et al., 2013), maize (Liang et al., 2014), sorghum (Jiang et al., 2013), wheat (Shan et al., 2013), etc. However, the majority of these studies relied on stable transformation by *Agrobacterium tumefaciens* to deliver the CRISPR vectors. As stable transformation of plants normally results in regeneration of mutation lines with integration of foreign DNA into the plant genome, this gene editing system raise regulatory concerns related to genetically modified plants in some countries (Woo et al., 2015).

Polyethylene glycol (PEG)-mediated protoplast transfection is an alternative for delivery of CRISPR vectors or ribonucleoprotein complexes (RNPs) into plant cells, which can produce transgene-free mutation lines through transient gene expression. However, as protoplast regeneration remains a bottleneck for many plant species, gene editing through the protoplast approach for trait improvement has not been widely applied in most of the crop species. Application of the protoplast approach for gene editing in crop species reported so far were mainly for research purpose (Nicolia et al., 2015; Woo et al., 2015; Malnoy et al., 2016; Kim et al., 2017; Liang et al., 2017; Lin et al., 2018), while in most cases no protoplast regeneration results were reported. Development of an efficient and reliable protoplast regeneration method is thus essential for the application of all currently available CRISPR gene editing systems for directly producing transgene-free mutants for many plant species.

Rapeseed is an important oil crop, accounting for about 16% of the total global vegetable oil production (USDA, 2019). Cultivated rapeseed is an allotetraploid species (*B. napus*; *2n* = 38, AACC) that was formed by polyploidization of two diploids ancestors, *B. oleracea* (genome CC) and *B. rapa* (genome AA) (Chalhoub et al., 2014). Although the gene editing system of CRISPR/Cas9 has been used in rapeseed for trait improvement, the published results so far relied on stable transformation with *Agrobacterium* (Braatz et al., 2017; Li et al., 2018; Huang et al., 2020; Zheng et al., 2020). To the best of our knowledge, only a few studies reported using protoplasts for gene editing by CRISPR/Cas9 in rapeseed, while none of them have reported success in obtaining mutation lines, i.e. no protoplast regeneration after transfection. Murovec et al. (2018) reported using RNPs for gene editing of rapeseed, but no mutations were detected after protoplast transfection. Lin et al. (2018) reported that the rapeseed genome could be mutated by CRISPR/Cas9 using the protoplast approach, but no regenerated plants from the transfected protoplasts were reported. All the published results indicate that proof-of-concept protoplast regeneration protocols for CRISPR/Cas9 genome editing are still lacking for most crop species in general, including rapeseed.

Development of protoplast culture technology in *Brassica* species started in the 1970s, and received a great amount of attention in the early 1980s for a variety of applications, including mutant isolation, somatic hybridization and genetic transformation. Although intensive studies on protoplast culture conditions were conducted, protoplast regeneration remained at very low levels in most cases. Furthermore, the regeneration frequency is often species and genotype dependent, making method improvement very challenging (Kielkowska and Adamus, 2012). This is mainly because a large number of conditions need to be optimized in order to obtain reasonably high regeneration frequencies for each species. These conditions include protoplast isolation method, protoplast density for culture, nutrients, type and concentration of sugars, concentrations and combinations of plant growth regulators (PGRs) in culture media, culture conditions and the developmental stage of protoplast calli capable of shoot induction, etc.

Apart from providing edible oil, rapeseed also contains a large amount of high quality protein, which remains in the seedcake after oil extraction. The seedcake is currently used only as animal feed due to the presence of antinutritional factors, which make the seedcake taste bitter and undesirable for food uses (Nour-Eldin et al., 2012). One of such antinutritional factors is glucosinolates (GSLs). GSLs are synthesized in vegetative tissues and transported to seeds in *Brassica* species, and this transport is mainly regulated by glucosinolate transporter (*GTR*) genes (Nour-Eldin et al., 2012). Eliminating or reducing the quantity of GSLs in seedcake is thus necessary to improve the rapeseed seedcake for feed and food uses.

In this study, we report a rapid and efficient protoplast transfection and regeneration protocol for rapeseed gene editing using CRISPR/Cas9. Using this protocol, we have obtained high transfection and mutation frequencies, and successfully obtained mutated plants with the targeted mutations in the *BnGTR* genes.

## Materials and Methods

### Plant Material

Seeds of spring rapeseed (*B. napus* L.) cv. Kumily, kindly provided by Lantmännen, Svalöf, Sweden, were used in this study.

### *In vitro* Culture Conditions

All *in vitro* cultures in this study were maintained in a controlled climate chamber which has a temperature of 23 °C/18 °C (day/night) and 16 h photoperiod with a light intensity of 40 μmol m^−2^ s^−1^ (cool white fluorescent tubes).

### Seed Germination

Seeds were surface sterilized using 15% (w/v) calcium hypochlorite (CaCl_2_O_2_) for 20 min, and then rinsed thoroughly with sterile water. Surface sterilized seeds were planted on germination medium in sterile plastic boxes. The germination medium contained half strength Murashige & Skoog (MS), 10 g l^−1^ sucrose, 7 g l^−1^ Bacto agar at pH 5.7. The boxes were placed in the climate chamber as stated above.

### Protoplast Isolation

Protoplasts were isolated according to Yoo et al. (2007), with some modifications. About 40 fully opened young leaves from 3–4 weeks old seedlings were sliced into fine pieces on wetted filter paper in a sterile Petri dish and incubated in plasmolysis solution (0.4 M mannitol at pH 5.7) for 30 min at room temperature (RT) in the dark. The leaf pieces were then treated with 10 ml enzyme solution and incubated for 14–16 h at RT in the dark with gentle shaking. The enzyme solution consisted of 1.5% (w/v) cellulase Onozuka^TM^ R-10 (Yakult Pharmaceutical Co., LTD., Tokyo, Japan), 0.6% (w/v) Macerozyme^TM^ R-10 (Yakult Pharmaceutical Co., Ltd.), 0.4 M mannitol, 10 mM MES, 0.1% (w/v) BSA, 1 mM CaCl_2_ and 1 mM β-mercaptoethanol at pH 5.7.

The isolated protoplasts were filtered through a 40 μm nylon cell strainer into a 50 ml Falcon tube, diluted with 30 ml W5 solution (Menczel et al., 1981) and centrifuged at 100 *g* for 10 min. Pellets were re-suspended in 10 ml W5 solution and centrifuged at 100 *g* for 5 min, and this process was repeated twice. Pellets were then re-suspended in 5 ml W5 solution and incubated on ice in the dark for 30 min. The supernatant was discarded and the protoplasts were diluted with 5–10 ml W5 solution based on the size of pellets. Protoplast solution of 15 μl was loaded on a hemocytometer for counting protoplasts under light microscope. After centrifugation for 3 min at 100 *g*, the protoplast density was adjusted to 400 000 to 600 000 per ml using 0.5 M mannitol solution. Equal volume of the protoplast suspension and alginate solution were mixed for making alginate disks. The alginate-solution consisted of 2.8% (w/v) sodium alginate and 0.4 M mannitol according to Kielkowska and Adamus (2012). To produce alginate disks, about 500 μl of the mixed protoplast and alginate suspension were pipetted onto the calcium-agar plates (0.4 M mannitol, 2.2 g l^−1^ CaCl_2_ and 10 g l^−1^ Phyto agar) and incubated at RT for 30 min. Thereafter, 2 ml of calcium-solution (50 mM CaCl_2_, 0.4 M mannitol) was added onto each disk and incubated for 1 h at RT to complete polymerization. The disks were then transferred to the culture medium as described below.

### Protoplast Culture in Liquid Medium

The prepared protoplast-alginate disks were cultured in 6-well tissue culture plates with one disk in each well and addition of 2–3 ml MI medium. Plates were covered with aluminium foil and kept at RT for 24 h, thereafter placed under fibre cloth without aluminium foil in the climate chamber under conditions as stated above. After 3–4 d, the MI medium was replaced by MII. MI medium consisted of 2.18 g l^−1^ Nitsch medium (Nitsch and Nitsch, 1969), 10 g l^−1^ sucrose, 10 g l^−1^ glucose, 100 g l^−1^ mannitol, 100 mg l^−1^ casein, 0.5 mg l^−1^ 2,4-dichlorophenoxyacetic acid (2,4-D) and 0.5 mg l^−1^ α-naphthaleneacetic acid (NAA) at pH 5.7. MII medium was the same as MI, but PGRs were changed to 1.1 mg l^−1^ thidiazuron (TDZ) and 0.05 mg l^−1^ 2,4-D instead. During this culture period, MII medium was renewed every 5–7 d.

### Plant Regeneration, Growth and Rooting on Solid Medium

After 20–25 d, the protoplast calli from the alginate disks were directly spread on the shoot induction medium (SIM) in Petri dishes for shoot induction. The SIM medium consisted of full-strength MS, 30 g l^−1^ sucrose, 50 g l^−1^ mannitol, 1.1 mg l^−1^ or 2.2 mg l^−1^ TDZ, 0.05 mg l^−1^ NAA, 0.5 mg l^−1^ AgNO_3_ and 2.5 g l^−1^ Gelrite at pH 5.7. After 10–20 d on the SIM medium, the protoplast calli were transferred to shoot regeneration medium (SRM) in Petri dishes for shoot regeneration. Different SRM media were designed, in which C- and N-sources, types and combinations of PGRs, as well as culture duration in MI, MII and on SIM medium were tested. The detailed experimental designs are presented in (Tables 1–7). The medium was renewed every 3–4 weeks during the shoot regeneration phase.

**Table 1:** Effect of PGRs in MI medium on protoplast growth and development of rapeseed.

**PGR conc. (mg l^**−1**^)**	Viability of protoplasts (%)^*^	**PGR conc.(mg l^**−1**^)**	Viability of protoplasts (%)^*^
TDZ 1.12,4-D 1.0	0.0 c	NAA 0.52,4-D 0.5	80.0 a
TDZ 1.12,4-D 0.5	0.0 c	BAP 2.0NAA 0.5	0.0 c
TDZ 1.12,4-D 0.25	0.0 c	Zeatin 1.0NAA 0.5	0.0 c
TDZ 0.552,4-D 0.5	0.0 c	BAP 2.02,4-D 0.5Zeatin 1.02,4-D 0.5	20.3 b 13.3 b

*Medium I composition: 2.18 g l^−1^ Nitsch medium, 10 g l^−1^ sucrose, 10 g l^−1^ glucose, 100 g l^−1^ mannitol, 100 mg l^−1^ casein at pH 5.7*.

* *Percentage of protoplasts maintained round and compact in form, and green in color, observed under light microscope 7 d after protoplast culture. Values followed by the same letter were not statistically different at p = 0.05 (n = 3)*.

**Table 2:** Effect of PGRs in MII medium on protoplast development of rapeseed.

**PGR conc. (mg l^**−1**^)**	Callus formation (%)^*^	**PGR conc.(mg l^**−1**^)**	Callus formation (%)^*^
BAP 1.0NAA 0.5	0.0 b	TDZ 1.1NAA 0.1	0.0 b
BAP 1.0NAA 0.1	0.0 b	TDZ 1.1NAA 0.05	75.0 a
BAP 2.0NAA 0.1	0.0 b	TDZ 1.12,4-D 0.1	0.0 b
TDZ 2.2NAA 0.1	0.0 b	TDZ 1.12,4-D 0.05	80.0 a

*The protoplasts were cultured in MI medium before being transferred to MII medium. Medium II composition: 2.18 g l^−1^ Nitsch medium, 10 g l^−1^ sucrose, 10 g l^−1^ glucose, 100 g l^−1^ mannitol, 100 mg l^−1^ casein at pH 5.7*.

* *The results were recorded when protoplast colonies were about 0.1 mm in diameter after 30 d in the MII medium. Values followed by the same letter were not statistically different at p = 0.05 (n = 3)*.

**Table 3:** Effect of PGRs in shoot induction medium (SIM) on protoplast regeneration of rapeseed.

**PGR conc. (mg l^**−1**^)**	Regeneration (%)^*^	**PGR conc. (mg l^**−1**^)**	Regeneration (%)^*^
TDZ 1.1NAA 0.05	0.0 b	TDZ 2.2NAA 0.05	0.0 b
TDZ 1.1NAA 0.05	35.0 a	TDZ 2.2NAA 0.05	40.0 a
Mannitol 50,000		Mannitol 50,000	

*SIM medium composition: Full strength MS, sucrose 30 g l^−1^, 0.5 mg l^−1^ AgNO_3_, 2.5 g l^−1^ Gelrite at pH 5.7*.

* *The results were recorded after one month on the SIM media. Values followed by the same letter were not statistically different at p = 0.05 (n = 3)*.

**Table 4:** Effect of PGRs in shoot regeneration medium (SRM) on protoplast regeneration of rapeseed.

**PGR conc. (mg l^**−1**^)**	Regeneration (%)^*^	**PGR conc.(mg l^**−1**^)**	**Regeneration (%)**
BAP 2.0NAA 0.1	0.0 c	Kinetin 2.0NAA 0.1	0.0 c
BAP 3.0NAA 0.2	0.0 c	TDZ 0.5NAA 0.1	0.0 c
BAP 5.0NAA 0.5	1.0 c	TDZ 1.1NAA 0.1	5.0 c
Zeatin 1.0NAA 0.1	0.0 c	TDZ 2.2NAA 0.5	45.0 a
Zeatin 2.0NAA 0.2	0.0 c	TDZ 2.2NAA 1.0	22.0 b

*SRM medium composition: Full strength MS, sucrose 20 g l^−1^, 0.5 mg l^−1^ AgNO_3_, 2.5 g l^−1^ Gelrite at pH 5.7*.

* *The results were recorded after one month on the SRM medium. Values followed by the same letter were not statistically different at p = 0.05 (n = 3)*.

**Table 5:** Effect of C-sources in shoot regeneration medium (SRM) on protoplast regeneration of rapeseed.

**Sugar conc. (g l^**−1**^)**	Regeneration (%)^*^	**Sugar conc. (g l^**−1**^)**	**Regeneration (%)**
Sucrose 15	30.6 b	Glucose 10	11.3 c
Sucrose 20	41.0 a	Glucose 20	10.0 c
Sucrose 30	31.4 ab		

*SRM composition: Full strength MS, 2.2 mg l^−1^ TDZ, 0.5 mg l^−1^ NAA, 0.5 mg l^−1^ AgNO_3_, 2.5 g l^−1^ Gelrite at pH 5.7*.

* *The results were recorded after one month on the SRM medium. Values followed by the same letter were not statistically different at p = 0.05 (n = 3)*.

**Table 6:** Effect of culture duration in MI and MII media on protoplast regeneration of rapeseed.

			**Regeneration**	(%)^*^			
**Duration**	**3d**	**5d**	**10d**	**15d**	**20d**	**30d**	**40d**
In MI	35.0 a	15.0 b	0.0 c	0.0 c	0.0 c	**-**	**-**
In MII	**-**	**-**	0.0 c	20.0 a	40.0 b	15.0 c	0.0 c

*Medium I composition: 2.18 g l^−1^ Nitsch medium, 10 g l^−1^ sucrose, 10 g l^−1^ glucose, 100 g l^−1^ mannitol, 100 mg l^−1^ casein, 2.2 mg l^−1^ NAA, 0.5 mg l^−1^ 2,4-D at pH 5.7. Medium II composition: 2.18 g l^−1^ Nitsch medium, 10 g l^−1^ sucrose, 10 g l^−1^ glucose, 100 g l^−1^ mannitol, 100 mg l^−1^ casein, 1.1 mg l^−1^ TDZ, 0.05 mg l^−1^ 2,4-D at pH5.7*.

* *The results were recorded after one month on the SRM medium, which consisted of full strength MS, 2.2 mg l^−1^ TDZ, 0.5 mg l^−1^ NAA, 0.5 mg l^−1^ AgNO_3_, 2.5 g l^−1^ Gelrite at pH 5.7. Values followed by the same letter were not statistically different at p = 0.05 (n = 3)*.

**Table 7:** Effect of culture duration on shoot induction medium (SIM) on protoplast regeneration of rapeseed.

			**Regeneration**	(%)^*^			
**Duration**	**15d**	**20d**	**25d**	**30d**	**40d**	**50d**	**60d**
SIM1	17.0 c	39.7 a	40.0 a	26.0 b	14.0 c	5.0 d	0.0 d
SIM2	17.0 bc	45.0 a	45.0 a	20.0 b	10.0 c	8.0 cd	0.0 d

*SIM1 composition: Full strength MS, 30 g l^−1^ sucrose, 50 g l^−1^ mannitol, 1.1 mg l^−1^ TDZ, 0.05 mg l^−1^ NAA, 0.5 mg l^−1^ AgNO_3_, 2.5 g l^−1^ Gelrite at pH 5.7. SIM2 composition: Full strength MS, 30 g l^−1^ sucrose, 50 g l^−1^ mannitol, 2.2 mg l^−1^ TDZ, 0.05 mg l^−1^ NAA, 0.5 mg l^−1^ AgNO_3_, 2.5 g l^−1^ Gelrite at pH 5.7*.

* *The results were recorded after two months on the SRM medium. Values followed by the same letter were not statistically different at p = 0.05 (n = 3)*.

The regenerated shoots were transferred to the shoot growing medium consisting of fullstrength MS, 20 g l^−1^ sucrose, 0.05 mg l^−1^ 6-benzyladnine (BAP), 0.03 mg l^−1^ gibberellic acid 3 (GA_3_) and Bacto agar 7.5 g l^−1^ at pH 5.7.

The elongated shoots were transferred to the rooting medium consisting of half strength MS, 20 g l^−1^ sucrose, 0.05 mg l^−1^ NAA and Bacto agar 7.5 g l^−1^ at pH 5.7. The rooted shoots were then planted in soil in the biotron with standard management. The growth conditions in the biotron were 21°C/16°C (day/night), 16 h photoperiod with a light intensity of 250 μmol m^−2^ s^−1^ and 60% humidity.

### Identification and Cloning of *GTR* Genes, sgRNA Design and Vector Construction

Two known *BnGTR* orthologs from *A. thaliana, AtGTR1* (*AT3G47960*) and *AtGTR2* (*AT5G62680*) were used for a BLAST query in the NCBI database against the rapeseed reference genome cv. ZS11 (Bra_napus_v2.0) and six paralogs of *BnGTR1* (*LOC106397267, LOC106408997, LOC106410496, LOC106414122, LOC106445255* and *LOC111202315*) and six paralogs of *BnGTR2* (*LOC106347844, LOC106366161, LOC106369007, LOC106405453, LOC106411192* and *LOC106424883*) were found (Table 8). Genomic and full-length open reading frames of six *BnGTR1* and six *BnGTR2* paralogs were amplified from genomic DNA and cDNA of cv. Kumily, respectively, using gene specific primers according to published protocols (Kim et al., 2020; Muthusamy et al., 2020), with minor modifications, and confirmed by sequencing. Genomic DNA sequences of different paralogs from the *BnGTR1* and *BnGTR2* were aligned to find conserved target sites among the paralogs of each gene. Based on the location in the target gene sequence, off target potential and the GC content, two target sequences for all six *BnGTR1* paralogs (one in exon 2 and one in exon 3) and two target sequences for all six *BnGTR2* paralogs (both in exon 2) (Table 9) were designed using CRISPR MultiTargeter (Prykhozhij et al., 2015). All the chosen target sequences were 20 bp and tested for their off-target potential in the rapeseed genome using Cas-Offinder (Bae et al., 2014). Each target sequence was integrated into a single guide RNA (sgRNA) expression cassette (Addgene plasmids# 66201, 66198, 66202, 66203) using the primers listed in Supplementary Table S1. Thereafter, all four sgRNA expression cassettes were sequentially ligated into the pYLCRISPR/Cas9P_ubi_-N vector according to the protocol described by Ma et al. (2015), resulting in a vector designated as pYLCRISPR/Cas9P_ubi_-GTR Supplementary Figure S1. Moreover, in order to examine if the transgene integration happened or not in the mutants, PCR was performed on the *Cas9* and *nptII* genes in the pYLCRISPR/Cas9P_ubi_-GTR vector using the gene specific primers (Supplementary Table S2). The PCR analyses were performed using Phusion High-Fidelity PCR Master Mix with GC Buffer (Thermo Scientific^TM^) according to the manufacturer's recommendations. The PCR conditions were 98°C for 3 min, followed by 30 cycles at 98°C for 10 s, 63°C for 30 s, 72°C for 30 s, with a final extension at 72°C for 8 min and the PCR products were separated on a 1% agarose gel.

**Table 8:** Features of the *BnGTR* paralogs used in this study.

**Arabidopsis orthologs**	***B. napus* genes**	**Locus number**	**Genomic sequence length (bp)**	**Number of exons**	**Coding region (bp)**
*AtGTR1*	*BnGTR1*	*LOC106397267*	2798	4	1905
	*BnGTR1*	*LOC106408997*	2673	4	1848
	*BnGTR1*	*LOC106410496*	2649	4	1848
	*BnGTR1*	*LOC106414122*	2666	4	1848
	*BnGTR1*	*LOC106445255*	2988	4	1905
	*BnGTR1*	*LOC111202315*	2685	4	1848
*AtGTR2*	*BnGTR2*	*LOC106347844*	2842	4	1839
	*BnGTR2*	*LOC106366161*	2755	4	1839
	*BnGTR2*	*LOC106369007*	8538	4	1839
	*BnGTR2*	*LOC106405453*	2453	4	1839
	*BnGTR2*	*LOC106411192*	2868	4	1836
	*BnGTR2*	*LOC106424883*	2754	4	1839

**Table 9:** CRISPR target sequences (sgRNAs).

**Name**	**Sequence (5^**′**^-3^**′**^)**	**Target gene**
sgRNA1	AATGAGACATTTGAGAAGAT	*BnGTR1*
sgRNA2	GAATCAACAGTTTCTTCAAC	*BnGTR1*
sgRNA3	TTTGAGAAGCTTGGGATCAT	*BnGTR2*
sgRNA4	TTCCTTTGCGACACTTACTT	*BnGTR2*

### Protoplast Transfection

For approximation of transfection efficiency, protoplasts were transfected with the vector pCW498-35S-GFiP-OcsT (14 743bp) harboring the *green fluorescent protein* gene (*GFP*) (Wood et al., 2009). For inducing mutations in the *BnGTR1* and *BnGTR2* genes, protoplasts were transfected with the pYLCRISPR/Cas9P_ubi_-GTR vector (18537 bp).

After isolating and washing protoplasts as described above, ~120 000 protoplasts were re-suspended in 200 μl freshly prepared MMG solution (0.5 M mannitol, 15 mM MgCl_2_, 4 mM MES) in a 2 ml Eppendorf tube. The solution was mixed with 40 μg pCW498-35S-GFiP-OcsT vector or pYLCRISPR/Cas9P_ubi_-GTR vector DNA and equal volume of freshly prepared PEG-calcium solution (25% (w/v) PEG 4000, 0.5 M mannitol and 0.1 M CaCl_2_). The reaction was stopped after 5 min by addition of 1.5 ml W5 and mixed by inversion of the tubes, followed by centrifugation at 100 g for 3 min and immediate removal of supernatant.

Protoplasts transfected with the pCW498-35S-GFiP-OcsT vector DNA were re-suspended in 1 ml MI, transferred to 12-well tissue culture plates and incubated in the dark at RT. The protoplasts transfected with the pYLCRISPR/Cas9P_ubi_-GTR vector DNA were re-suspended in 200 μl 0.5 M mannitol and embedded in alginate disks as described above.

### Detection of *GFP* Gene Expression and Identification of *BnGTR* Mutants

For estimation of transfection efficiency, the protoplasts transfected with the *GFP* vector were observed after 48 h with Zeiss LSM 880 Airyscan confocal laser scanning microscope using an EC-Plan-Neofluar 10x/0.30 M27 objective for validation of GFP expression. Excitation wavelength was 488 nm and detection wavelength was 490–585 nm. Non-transfected protoplasts were used as control to verify that no auto-fluorescence could be observed.

To identify mutations in the *BnGTR* genes, genomic DNA was extracted from the regenerated shoots using Phire Plant Direct PCR kit (Thermo Scientific^TM^) and used as template for PCR amplification of the target sequences with fluorescently labeled forward primers using Phusion High-Fidelity PCR Master Mix with GC Buffer (Thermo Scientific^TM^) (Supplementary Table S3). The PCR amplicons were subjected to high-resolution fragment analysis (HRFA) as described by Andersson et al. (2017). For confirmation of the mutations by sequencing, PCR amplicons with non-labeled primers were ligated into the pJET1.2/blunt cloning vector (Thermo Scientific^TM^) and transformed into Stellar^TM^ chemically competent cells of *E. coli* (Takara Bio, Shiga, Japan). Randomly selected single colonies were analyzed by Sanger sequencing (Eurofins Genomics, Konstanz, Germany).

### Statistical Analysis

For the protoplast viability test, protoplast solution was loaded on a Hemocytometer and five 1 mm^2^ squares were observed under light microscope seven days after culture, and this was repeated three times. For the callus and shoot regeneration tests, each treatment consisted of 40–50 protoplast colonies, and was repeated three times. The regeneration results were recorded about 1–3 months after shoots started to appear, depending on experiment. The detailed information is presented at the bottom of each corresponding table in the result section. Data was analyzed with ANOVA and Tukey's test using the statistical software Minitab (LLC) version 19.2020.1.

## Results

### Effect of PGRs in MI Medium on Protoplast Viability at the Initial Stage

**Figure 1:**
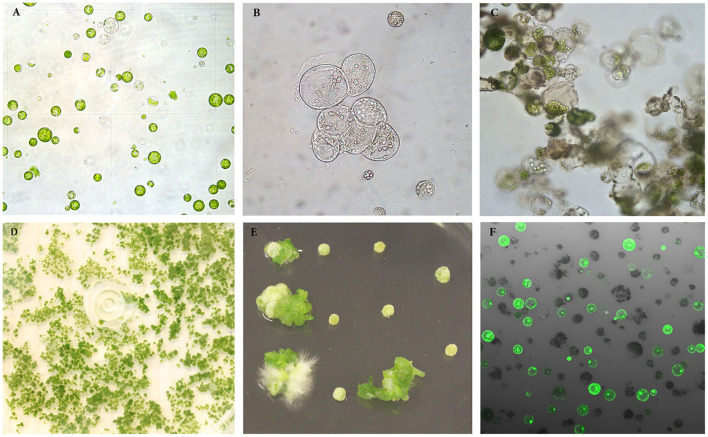
Isolation, regeneration and transfection of protoplasts of rapeseed. **(A)** Freshly isolated protoplasts. **(B, C)** Protoplasts undergoing cell divisions and multiplication. **(D)** Protoplast colonies. **(E)** Shoot regeneration from protoplast colonies. **(F)** Transfected protoplasts expressing GFP protein observed under confocal laser scanning microscope.

Protoplasts are very fragile and sensitive to the growth environment when they are freshly isolated due to lacking the cell wall. The medium composition, especially PGRs, is crucial to the initial protoplast culture. We thus tested several PGR combinations in MI medium, and found that the combination of 0.5 mg l^−1^ 2,4-D and 0.5 mg l^−1^ NAA gave the best result in terms of protoplast viability among all PGR combinations tested. In this medium, most protoplasts remained viable, as they were observed under a light microscope to be round and compact in form and green in color (Figure 1A) 7 d after protoplast culture. The protoplasts in the MI medium containing other PGR combinations became inviable (Table 1), namely shrunk and pale or brownish in color. This result is in agreement with the results from a previous report, which indicated that 2,4-D was essential for cell wall formation and initial protoplast growth (Glimelius, 1984). Moreover, our results showed that addition of cytokinin, like TDZ, BAP or zeatin, in combination with auxin in MI medium did not improve protoplast viability or growth compared with auxin alone.

### Effect of PGRs in MII Medium on Protoplast Growth and Development

After the cell wall has formed, the protoplasts would undergo a rapid cell division (Figures 1B, C), and a suitable PGR combination in MII medium was found to be essential during this stage. We investigated different PGR combinations in MII medium. The results showed that the combinations of 1.1 mg l^−1^ TDZ with 0.05 mg l^−1^ 2,4–D and 1.1 mg l^−1^ TDZ with 0.05 mg l^−1^ NAA gave better results than the other PGR combinations tested, as the protoplasts divided rapidly and formed multiple protoplast colonies on these two media (Table 2), indicating that a relatively lower concentration of auxin was necessary for protoplast growth and further development during this stage. The results also showed that TDZ as cytokinin source was much more efficient than BAP for facilitating the normal growth of the protoplasts.

### Effect of Mannitol in SIM Medium on Protoplast Regeneration

Our results showed that culture of the protoplasts in MII medium longer than 20 d would result in brownish and inviable protoplasts (Table 6), likely due to inhibitory effect of high concentration of mannitol (100 g l^−1^) on growth. To solve this problem, the protoplasts were transferred to SIM medium after 20 d, which contained half amount of mannitol compared to MII. As shown in (Table 3), the presence of mannitol in the SIM medium was still necessary for callus growth (Figure 1D), and thereby facilitating shoot regeneration (Figure 1E). Otherwise, the calli could become brownish, and no shoot regeneration would occur. This suggests that osmotic protection by mannitol was needed for maintaining the normal growth and development of protoplasts during this stage of protoplast culture.

### Effect of PGRs in SRM Medium on Protoplast Regeneration

In this study, we found that the combination of TDZ as cytokinin-source and NAA as auxin-source in SRM medium gave the best result with regards to shoot regeneration among all the combinations tested (Table 4). Relatively high concentrations of cytokinin and auxin gave better effect on shoot regeneration, in which 2.2 mg l^−1^ TDZ in combination with 0.5 mg l^−1^ NAA gave the highest regeneration frequency, while all other PGR combinations resulted in a significantly decreased regeneration frequency.

### Effect of C-Source in SRM Medium on Protoplast Regeneration

Sugar plays an important role in protoplast growth and development. We tested two types of sugars commonly used in protoplast culture as carbon source in the SRM media. The results showed that sucrose resulted in better shoot regeneration frequency than glucose, which seemed to be less effective in promoting shoot regeneration (Table 5). When comparing different concentrations of sucrose, we found that 20 g l^−1^ sucrose resulted in 41.0% regeneration frequency after two months, compared to 31.4% for 30 g l^−1^.

### Effect of Culture Duration in MI, MII and SIM Media on Protoplast Growth and Regeneration

We found that the culture duration in MI and MII media at the early stage of protoplast development was critical for shoot regeneration. The results in (Table 6) show that the culture duration in MI medium should not be longer than 5 d, while 15–20 d in MII medium was the most suitable duration for shoot regeneration. After 30 d in MII medium, the regeneration percentage decreased rapidly.

The culture duration on SIM medium also seemed to be important for shoot regeneration, as shown in (Table 7). The duration of 20–25 d on SIM medium was shown to be the most suitable duration among all durations tested for shoot regeneration. After 30 d, the regeneration percentage was significantly decreased.

### Cloning of *BnGTR* Paralog Genes

All the 12 paralogs were amplified in cv. Kumily in this study, and the gene sequences were submitted in the GenBank database under the accession numbers, MW759464 to MW759475. The homology between different paralogs of the same gene family ranged between 86% to 99% for *BnGTR1* and 88% to 98% for *BnGTR2*.

### Protoplast Transfection Efficiency

In order to estimate the efficiency of protoplast transfection, we transfected protoplasts with a vector harboring the *GFP* gene. The results showed that transfection efficiencies ranging from approximately 40 to 80% could routinely be observed, as measured by intact protoplasts exhibiting GFP fluorescence (Figure 1F) 48 h after transfection. This suggests that a large proportion of the protoplasts can express the transgene for a sustained time-period, and that the transfection protocol is working well for rapeseed under our culture conditions.

### Identification of Mutation in the *BnGTR* Genes

**Figure 2:**
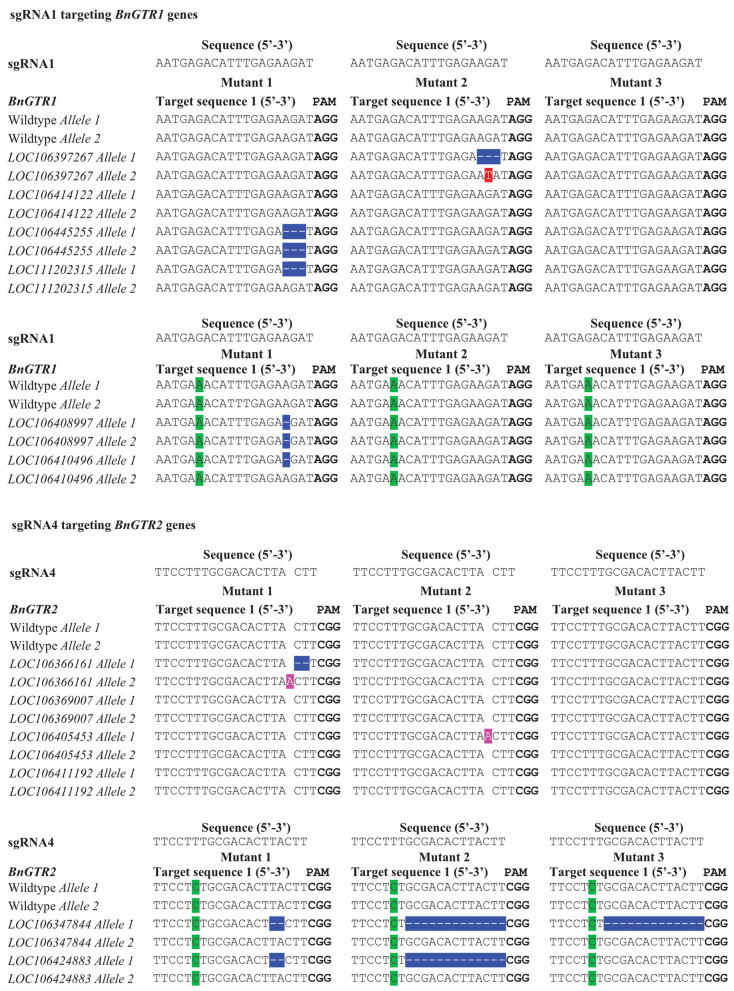
Types of mutations in the *BnGTR1* and *BnGTR2* genes detected in the three mutants in comparison with wild type of rapeseed cv. Kumily, determined by DNA sequencing. PAM sites are highlighted in bold letters. Mismatches with the sgRNAs are highlighted in green. Mutated nucleotides were highlighted in different colors, in which deletions are shown with hyphens in blue, substitution and insertions are highlighted in red and pink, respectively.

We designed four highly conserved 20 bp target sequences (sgRNAs) for *BnGTR1* (sgRNA1 and sgRNA2) and *BnGTR2* (sgRNA3 and sgRNA4), for knocking out all paralogs of the two gene families. The sgRNA1 and sgRNA4 sequences shared 100% identity with the target regions in four paralogs of *BnGTR1* and *BnGTR2*, but had a single nucleotide mismatch 14 bp upstream of the PAM site in two paralogs of each targeted gene family (Figure 2). The sgRNA2 and sgRNA3 sequences had 100% identity in five paralogs of *BnGTR1* and *BnGTR2*, but had a single nucleotide mismatch 12 bp upstream of the PAM site in one paralog of *BnGTR1* and one nucleotide mismatch in one paralog of *BnGTR2* 17 bp upstream of the PAM site.

Using the above optimized protoplast regeneration and transfection protocols and the CRISPR vector harboring the four sgRNAs, we have successfully mutated multiple *BnGTR* genes.

Out of 50 calli, 16 shoots were regenerated, resulting in a regeneration frequency of over 30%. Out of the 16 regenerated shoots, 3 were found to be mutated, giving a mutation efficiency of over 18%. The results were based on three biological replicates. The sequencing results revealed various types of mutations consisting of single base insertions, 1-13 bp deletions and a substitution among the three mutant lines analyzed (Figure 2), indicating successful gene editing using our optimized protoplast protocol. No mutations at the target sites of sgRNA2 and sgRNA3 were detected. Sequencing results revealed that the mutations in deletion and insertion could lead to frameshift mutations and introduce premature stop codons to disrupt the open-reading frames. The PCR results showed no presence of the *Cas9* and *nptII* genes in the three mutants (Supplementary Figure S2).

## Discussion

Creation of more genetic variation is necessary to improve important agronomic traits of rapeseed, as the natural gene pool of the species has a low genetic diversity (Bus et al., 2011). Apart from crossbreeding, induced mutations has been used to increase genetic variation of the species. In recent years, the CRISPR/Cas9 technology has been proven to be a powerful tool for plant genetic modification, while its great potential has not been explored fully yet for trait improvement, and this is particularly true for rapeseed. One of the main reasons for this is the lack of an efficient method for delivering CRISPR vectors or complexes into plant cells for production of transgene-free mutation lines. The protoplast transient transfection system is a promising approach for delivering CRISPR complexes, but the bottleneck of this approach is the difficulty in protoplast regeneration.

Protoplasts are plant cells that lack the cell wall, but possess plasma membrane and all other cellular components. The first developmental stage of protoplasts is formation of the cell wall, followed by cell divisions. The cell wall formation starts within a few hours after isolation, and may take several days to complete (Kartha et al., 1974). In this period, the protoplasts are very fragile and sensitive to the culture conditions and surrounding environment. It has been reported that for the culture of rapeseed hypocotyl protoplasts, the auxins 2,4-D and NAA were both necessary for cell wall formation and cell division. The ratio of NAA to 2,4-D content that stimulates protoplast colony growth best appears to be species- and even genotype-dependent. It has been reported that, in one case, a higher level of NAA than 2,4-D was either similar or better in stimulating protoplast colony growth of all genotypes tested (Glimelius, 1984), while in another study, higher levels of 2,4-D than NAA was reported to be beneficial for hypocotyl protoplast development in rapeseed (Barsby et al., 1986). In this study, we used identical quantities of 2,4-D and NAA, and it turned out to work well in this case.

Osmotic pressure must be maintained at the initial stage of protoplast culture. The isolated and cultured protoplasts require osmotic protection until they have developed cell walls (Kao and Seguin-Swartz, 1987), while the osmolarity should be gradually reduced to a normal level in order to maintain normal growth and development. In this study, mannitol was used to maintain osmotic pressure. We first used a high concentration of mannitol (100 g l^−1^) in MI and MII media, which was then reduced to 50 g l^−1^ in SIM until the protoplasts became small colonies, and thereafter removed completely in the SRM medium. If mannitol was removed from the medium too early, the protoplasts would become brownish and eventually die. On the other hand, if the mannitol was removed from medium too late, the growth and regeneration of protoplasts would be negatively affected. The reason could be that continuous presence of mannitol would form an inappropriate cell environment for normal growth, e.g., affecting negatively the uptake of nutrients and water.

The culture density of protoplasts is also an important factor affecting protoplast growth and development. Some studies suggested that higher culture densities would promote the growth and division of protoplast cells (Chuong et al., 1985; Kielkowska and Adamus, 2012). The reason for this could be that cultured protoplasts stimulate growth and mitotic division of adjacent cells by releasing growth factors into the surrounding medium (Davey et al., 2005). In this study, we also found that a low density of protoplasts could result in poor cell division and thus reduced callus formation. However, too high density of protoplasts would result in brownish colonies, likely because of rapidly depleted available nutrients that caused a large number of protoplasts to fail to undergo divisions (Chuong et al., 1985). The most suitable plating density in this study was 0.4 million protoplasts per ml for rapeseed, while up to 1 million per ml also lead to regeneration of plants in many cases.

Low regenerative capacity is the major obstacle affecting the application of protoplasts for rapeseed. With induction and appropriate manipulations, the protoplasts are able to undergo a series of differentiation stages, and finally form whole plants under optimal or suitable conditions. Among all factors affecting protoplast regeneration, PGRs is thought to be the most important one. A general concept is that high auxin to cytokinin ratio is suitable to stimulate cell divisions and cell wall formation of protoplasts, and high cytokinin to auxin ratio is required for shoot regeneration. However, this ratio varies a lot from species to species (Kao and Seguin-Swartz, 1987), and thus needs to be optimized for each crop. We found in our study that TDZ gave the best shoot regeneration among all types of cytokinin tested. Moreover, high concentration of cytokinin in combination with a relatively high level of auxin (2.2 mg l^−1^ TDZ and 0.5 mg l^−1^ NAA) had a great positive effect on protoplast regeneration in rapeseed. Although BAP is widely used for many crops for *in vitro* cultures, it did not seem to be effective for protoplast regeneration in rapeseed, as shown in this study.

We also found in this study that the culture duration in different culture media at different developmental stages played an important role in protoplast regeneration of rapeseed, in which prolonged culture durations at earlier stages of development would reduce regeneration rapidly. For instance, the culture duration in MI medium should not be longer than 5 d, the duration in MII should be shorter than 30 d and not more than 20 d in SIM medium. These findings suggest that it is crucial to transfer protoplast cultures into the successive media in a timely manner.

In this study, the *BnGTR* genes were successfully edited by CRISPR/Cas9 in rapeseed using our optimized protoplast regeneration and transfection protocols, demonstrating for the first time the high capacity of the protoplast approach in genetic improvement of rapeseed by CRISPR/Cas9. We believe that this optimized protoplast regeneration protocol will be beneficial to other researchers working with rapeseed or other *Brassica* species. We are still working on generating more mutation lines in order to get desirable and more homozygous mutation lines. It should be kept in mind that modern widely cultivated cultivars are allotetraploid. This allopolyploidization leads to multiple homologs of most genes controlling the same traits in the rapeseed genome compared with the related diploid model species *A. thaliana* (Chalhoub et al., 2014). In order to develop a knockout mutant in rapeseed, it is imperative to edit all paralogous sequences of the *BnGTR* genes. Therefore, selfing for a couple of generations might be needed to obtain homozygous mutation lines in all paralogs of the *BnGTR* genes.

## Data Availability Statement

The original contributions presented in the study are included in the article/Supplementary Material, further inquiries can be directed to the corresponding author/s.

## Author Contributions

L-HZ led the research and, together with XL and SK, designed the studies. XL, SS, and SK performed the most of the experiments. XL, L-HZ, SK, and SS wrote the manuscript. OM, RG, EI, and EW contributed to the protoplast transfection and regeneration studies. All authors have read the manuscript and approved the submitted version.

## Conflict of Interest

The authors declare that the research was conducted in the absence of any commercial or financial relationships that could be construed as a potential conflict of interest.

## References

[B1] Andersson M. Turesson H. Nicolia A. Falt A. S. Samuelsson M. Hofvander P. (2017). Efficient targeted multiallelic mutagenesis in tetraploid potato (*Solanum tuberosum*) by transient CRISPR-Cas9 expression in protoplasts. Plant Cell Rep. 36, 117–128. 10.1007/s00299-016-2062-3 27699473 PMC5206254

[B2] Arora L. Narula A. (2017). Gene editing and crop improvement using CRISPR-Cas9 system. Front Plant Sci. 8:1932. 10.3389/fpls.2017.01932 29167680 PMC5682324

[B3] Bae S. Park J. Kim J. S. (2014). Cas-OFFinder: a fast and versatile algorithm that searches for potential off-target sites of Cas9 RNA-guided endonucleases. Bioinformatics 30, 1473–1475. 10.1093/bioinformatics/btu048 24463181 PMC4016707

[B4] Barsby T. L. Yarrow S. A. Shepard J. F. (1986). A rapid and efficient alternative procedure for the regeneration of plants from hypocotyl protoplasts of *Brassica napus*. Plant Cell Rep. 5, 101–103. 10.1007/BF00269244 24248044

[B5] Braatz J. Harloff H. J. Mascher M. Stein N. Himmelbach A. Jung C. (2017). CRISPR-Cas9 targeted mutagenesis leads to simultaneous modification of different homoeologous gene copies in polyploid oilseed rape (*Brassica napus*). Plant Physiol. 174, 935–942. 10.1104/pp.17.00426 28584067 PMC5462057

[B6] Bus A. Korber N. Snowdon R. J. Stich B. (2011). Patterns of molecular variation in a species-wide germplasm set of *Brassica napus*. Theor. Appl. Genet. 123, 1413–1423. 10.1007/s00122-011-1676-7 21847624

[B7] Chalhoub B. Denoeud F. Liu S. Parkin I. a,. P Tang H. Wang X.. (2014). Early allopolyploid evolution in the post-neolithic *Brassica napus* oilseed genome. Science 345, 950–953. 10.1126/science.1253435 25146293

[B8] Chuong P. V. Pauls K. P. Beversdorf W. D. (1985). A simple culture method for *Brassica* hypototyl protoplasts. Plant Cell Rep. 4, 4–6. 10.1007/BF00285492 24253633

[B9] Davey M. R. Anthony P. Power J. B. Lowe K. C. (2005). Plant protoplasts: status and biotechnological perspectives. Biotechnol. Adv. 23, 131–171. 10.1016/j.biotechadv.2004.09.008 15694124

[B10] Glimelius K. (1984). High growth rate and regeneration capacity of hypocotyl protoplasts in some Brassicaceae. Physiol. Plant 61, 38–44. 10.1111/j.1399-3054.1984.tb06097.x

[B11] Huang H. Cui T. Zhang L. Yang Q. Yang Y. Xie K.. (2020). Modifications of fatty acid profile through targeted mutation at *BnaFAD2* gene with CRISPR/Cas9-mediated gene editing in *Brassica napus*. Theor. Appl. Genet. 133, 2401–2411. 10.1007/s00122-020-03607-y 32448919

[B12] Jiang W. Zhou H. Bi H. Fromm M. Yang B. Weeks D. P. (2013). Demonstration of CRISPR/Cas9/sgRNA-mediated targeted gene modification in arabidopsis, tobacco, sorghum and rice. Nucleic Acids Res. 41:e188. 10.1093/nar/gkt780 23999092 PMC3814374

[B13] Kao H. M. Seguin-Swartz G. (1987). Study of factors affecting the culture of *Brassica napus* L. and *B. juncea Coss*. mesophyll protoplasts. Plant Cell Tissue Organ Cult. 10, 79–90. 10.1007/BF00035906

[B14] Kartha K. K. Michayluk M. R. Kao K. N. Gamborg O. L. Constabel F. (1974). Callus formation and plant regeneration from mesophyll protoplasts of rape plants (*Brassica napus* L. cv. Zephyr). Plant Sci. Lett. 3, 265–271. 10.1016/0304-4211(74)90097-2

[B15] Kielkowska A. Adamus A. (2012). An alginate-layer technique for culture of *Brassica oleracea* L. protoplasts. In Vitro Cell Dev. Biol. Plant 48, 265–273. 10.1007/s11627-012-9431-6 22593638 PMC3337407

[B16] Kim H. Kim S. T. Ryu J. Kang B. C. Kim J. S. Kim S. G. (2017). CRISPR/Cpf1-mediated DNA-free plant genome editing. Nat. Commun. 8:14406. 10.1038/ncomms14406 28205546 PMC5316869

[B17] Kim S.-Y. Bengtsson T. Olsson N. Hot V. Zhu L.-H. Åhman I. (2020). Mutations in two aphid-regulated β-1,3-glucanase genes by CRISPR/Cas9 do not increase barley resistance to *Rhopalosiphum padi* L. Front. Plant Sci. 11:1403. 10.3389/fpls.2020.01043 32754185 PMC7381296

[B18] Li C. Hao M. Wang W. Wang H. Chen F. Chu W.. (2018). An efficient CRISPR/Cas9 platform for rapidly generating simultaneous mutagenesis of multiple gene homoeologs in allotetraploid oilseed rape. Front. Plant Sci. 9:442. 10.3389/fpls.2018.00442 29731757 PMC5920024

[B19] Liang Z. Chen K. Li T. Zhang Y. Wang Y. Zhao Q.. (2017). Efficient DNA-free genome editing of bread wheat using CRISPR/Cas9 ribonucleoprotein complexes. Nat. Commun. 8:14261. 10.1038/ncomms14261 28098143 PMC5253684

[B20] Liang Z. Zhang K. Chen K. Gao C. (2014). Targeted mutagenesis in *Zea mays* using TALENs and the CRISPR/Cas system. J. Genet. Genomics 41, 63–68. 10.1016/j.jgg.2013.12.001 24576457

[B21] Lin C. S. Hsu C. T. Yang L. H. Lee L. Y. Fu J. Y. Cheng Q. W.. (2018). Application of protoplast technology to CRISPR/Cas9 mutagenesis: from single-cell mutation detection to mutant plant regeneration. Plant Biotechnol. J. 16, 1295–1310. 10.1111/pbi.12870 29230929 PMC5999315

[B22] Ma X. Zhang Q. Zhu Q. Liu W. Chen Y. Qiu R.. (2015). A robust CRISPR/Cas9 system for convenient, high-efficiency multiplex genome editing in monocot and dicot plants. Mol. Plant 8, 1274–1284. 10.1016/j.molp.2015.04.007 25917172

[B23] Malnoy M. Viola R. Jung M. H. Koo O. J. Kim S. Kim J. S.. (2016). DNA-free genetically edited grapevine and apple protoplast using CRISPR/Cas9 ribonucleoproteins. Front. Plant Sci. 7:1904. 10.3389/fpls.2016.01904 28066464 PMC5170842

[B24] Menczel L. Nagy F. Kiss Z. R. Maliga P. (1981). Streptomycin resistant and sensitive somatic hybrids of *Nicotiana tabacum* + *Nicotiana knightiana*: correlation of resistance to *N. tabacum* plastids. Theor. Appl. Genet. 59, 191–195. 10.1007/BF00264975 24276446

[B25] Murovec J. Gucek K. Bohanec B. Avbelj M. Jerala R. (2018). DNA-free genome editing of *Brassica oleracea* and *B. rapa* protoplasts using CRISPR-Cas9 ribonucleoprotein complexes. Front. Plant Sci. 9:1594. 10.3389/fpls.2018.01594 30455712 PMC6230560

[B26] Muthusamy S. Vetukuri R. R. Lundgren A. Ganji S. Zhu L.-H. Brodelius P. E.. (2020). Transient expression and purification of β-caryophyllene synthase in *Nicotiana benthamiana* to produce β-caryophyllene *in vitro*. PeerJ 8:e8904. 10.7717/peerj.8904 32377446 PMC7194099

[B27] Nekrasov V. Staskavicz B. Wigel D. Jones J. D. G. Kamoun S. (2013). Targeted mutagenesis in the model plant *Nicotiana benthamiana* using Cas9 RNA-guided endonuclease. Nat. Biotechnol. 31, 691–693. 10.1038/nbt.2655 23929340

[B28] Nicolia A. Proux-Wera E. Ahman I. Onkokesung N. Andersson M. Andreasson E.. (2015). Targeted gene mutation in tetraploid potato through transient TALEN expression in protoplasts. J. Biotechnol. 204, 17–24. 10.1016/j.jbiotec.2015.03.021 25848989

[B29] Nitsch J. P. Nitsch C. (1969). Haploid plants from pollen grains. Science 163, 85–87. 10.1126/science.163.3862.85 17780179

[B30] Nour-Eldin H. H. Andersen T. G. Burow M. Madsen S. R. Jorgensen M. E. Olsen C. E.. (2012). NRT/PTR transporters are essential for translocation of glucosinolate defence compounds to seeds. Nature 488, 531–534. 10.1038/nature11285 22864417

[B31] Prykhozhij S. V. Rajan V. Gaston D. Berman J. N. (2015). CRISPR multitargeter: a web tool to find common and unique CRISPR single guide RNA targets in a set of similar sequences. PLoS ONE 10:e0119372. 10.1371/journal.pone.0119372 25742428 PMC4351176

[B32] Shan Q. Wang Y. Li J. Zhang Y. Chen K. Liang Z.. (2013). Targeted genome modification of crop plants using a CRISPR-Cas system. Nat. Biotechnol. 31, 686–688. 10.1038/nbt.2650 23929338

[B33] USDA (2019). Oil Crops Yearbook. Available online at: https://www.ers.usda.gov/data-products/oil-crops-yearbook/ (accessed April 19, 2019).

[B34] Woo J. W. Kim J. Kwon S. I. Corvalan C. Cho S. W. Kim H.. (2015). DNA-free genome editing in plants with preassembled CRISPR-Cas9 ribonucleoproteins. Nat. Biotechnol. 33, 1162–1164. 10.1038/nbt.3389 26479191

[B35] Wood C. C. Petrie J. R. Shrestha P. Mansour M. P. Nichols P. D. Green A. G.. (2009). A leaf-based assay using interchangeable design principles to rapidly assemble multistep recombinant pathways. Plant Biotechnol. J. 7, 914–924. 10.1111/j.1467-7652.2009.00453.x 19843252

[B36] Yoo S. D. Cho Y. H. Sheen J. (2007). Arabidopsis mesophyll protoplasts: a versatile cell system for transient gene expression analysis. Nat. Protoc. 2, 1565–1572. 10.1038/nprot.2007.199 17585298

[B37] Zheng M. Zhang L. Tang M. Liu J. Liu H. Yang H.. (2020). Knockout of two *BnaMAX1* homologs by CRISPR/Cas9-targeted mutagenesis improves plant architecture and increases yield in rapeseed (*Brassica napus* L.). Plant Biotechnol. J. 18, 644–654. 10.1111/pbi.13228 31373135 PMC7004912

