# Corrigendum: Efficient protoplast regeneration protocol and CRISPR/Cas9-mediated editing of glucosinolate transporter (*GTR*) genes in rapeseed (*Brassica napus* L.)

**DOI:** 10.3389/fpls.2023.1183684

**Published:** 2023-03-22

**Authors:** Xueyuan Li, Sjur Sandgrind, Oliver Moss, Rui Guan, Emelie Ivarson, Eu Sheng Wang, Selvaraju Kanagarajan, Li-Hua Zhu

**Affiliations:** Department of Plant Breeding, Swedish University of Agricultural Sciences, Lomma, Sweden

**Keywords:** *Brassica napus*, CRISPR/Cas9, gene editing, glucosinolate transporter, *GTR* gene, protoplast regeneration

In the published article, there was an error in the Funding statement. The Funding statement “FORMAS -The Swedish Research Council for sustainable development” missed the parentheses for the full name of FORMAS and grant numbers. The correct statement is “FORMAS (The Swedish Research Council for sustainable development) grants 2016-01401 and 2018-01301”.

The correct Funding statement appears below.

