# Supplementary material for: Efficient Protoplast Regeneration Protocol and CRISPR/Cas9-Mediated Editing of Glucosinolate Transporter (GTR) Genes in Rapeseed (Brassica napus L.)
Source: Front Plant Sci. 2021 Jul 7;12:680859. doi: 10.3389/fpls.2021.680859 (PMC8294089; doi:10.3389/fpls.2021.680859)
Supplement: Supplementary file 1 [file Presentation_1.pdf]

SUPPLEMENTARY MATERIAL

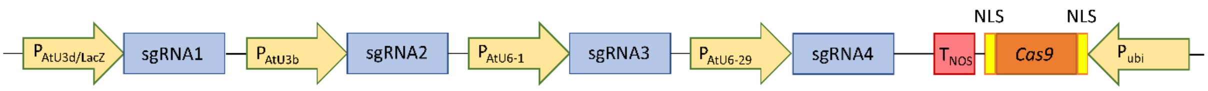

Supplementary Figure S1. Schematic representation of the CRISPR/Cas9 vector, pYLCRISPR/Cas9Pubi-GTR containing four sgRNA expression cassettes, designed for mutagenesis of the six *BnGTR1* and six *BnGTR2* paralogs in rapeseed. The sgRNA1, driven by *A. thaliana* U3d promoter along with *LacZ* gene as a cloning selection marker; the sgRNA2 driven by *A. thaliana* U3b promoter; the sgRNA3, driven by *A. thaliana* U6–1 promoter; the sgRNA4, driven by *A. thaliana* U6–29 promoter.

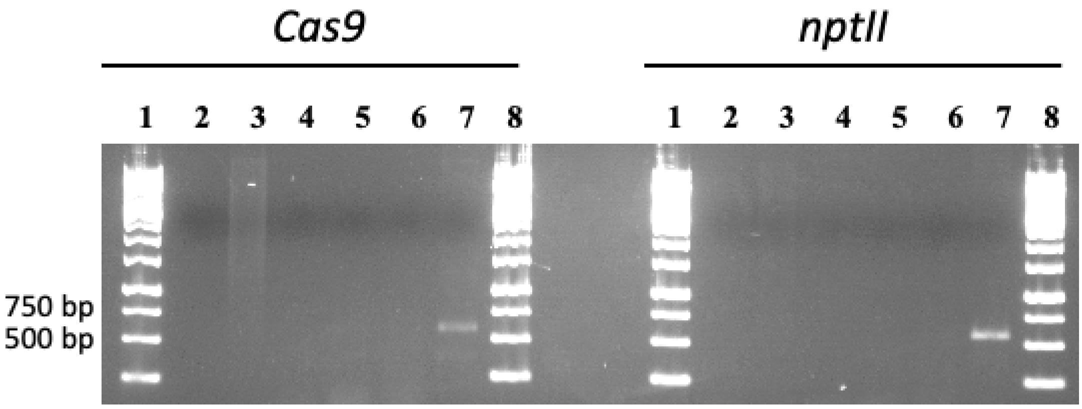

Supplementary Figure S2. | PCR analysis of the *Cas9*- and *nptII* genes in the mutants using the gene specific primers. Lane 1, 1kb ladder; lane 2, negative control (no template); lane 3, wildtype; lane 4, mutant 1; lane 5, mutant 2; lane 6, mutant 3; lane 7, positive control (pYLCRISPR/Cas9P$_{ubi}$-GTR vector); lane 8, 1kb ladder. The PCR product was 567 bp for *Cas9* and 630 bp for *nptII*.

Supplementary Table S1. List of primers used for expression cassette construction

Primer Name	Plasmid	Sequence (5’-3’)	Description
BnFP1	pYLsgRNA-AtU3d/LacZ	GTCAATGAGACATTTGAGAAGAT	sgRNA1-GTR1
BnRP1	pYLsgRNA-AtU3d/LacZ	AAACATCTTCTCAAATGTCTCAT	sgRNA1-GTR1
BnFP2	pYLsgRNA-AtU3b	GTCAATGAAACATTTGAGAAGAT	sgRNA2-GTR1
BnRP2	pYLsgRNA-AtU3b	AAACATCTTCTCAAATGTTTCAT	sgRNA2-GTR1
BnFP3	pYLsgRNA-AtU6-1	ATTGAATCAACAGTTTCTTCAAC	sgRNA3-GTR2
BnRP3	pYLsgRNA-AtU6-1	AAACGTTGAAGAAACTGTTGATT	sgRNA3-GTR2
BnFP4	pYLsgRNA-AtU6-29	ATTGAATCAATAGTTTCTTCAAC	sgRNA4-GTR2
BnRP4	pYLsgRNA-AtU6-29	AAACGTTGAAGAAACTATTGATT	sgRNA4-GTR2

Supplementary Table S2. | List of primers used in PCR for transgene detection

Primer Name	Gene	Sequence (5’-3’)
Cas9 FOR	*Cas9*	CTGCTTCCATGATCAAGCGC
Cas9 REV	*Cas9*	CCTTCTCGTTGGGGAGGTTC
nptII FOR	*nptII*	CTATTCGGCTATGACTGGGC
nptII REV	*nptII*	AATATCACGGGTAGCCAACG

Supplementary Table S3. List of primers used in HRFA analysis

Primer Name	Gene	Sequence (5'-3')
BnGTR1 LOC106397267-LOC106445255 F FAM	BnaA06g20740D BnaCnng63460D	GTTGTTACTTTATGGTTTGACT
BnGTR1 LOC106397267-LOC106445255 R	BnaA06g20740D BnaCnng63460D	AAATGACGCAGGCCAAGAA
BnGTR1 LOC106414122-LOC111202315 F HEX	BnaA01g20270D BnaC01g25280D	TCTTGTCACGTTGGCTTGAC
BnGTR1 LOC106414122-LOC111202315 R	BnaA01g20270D BnaC01g25280D	TGAGCGAGATGATCTGCGCG
BnGTR1 LOC106408997-LOC106410496 F PET	BnaC03g75950D BnaA06g16980D	CTCAACACGGTCCAGAAACT
BnGTR1 LOC106414122-LOC111202315 R	BnaC03g75950D BnaA06g16980D	TGAGCGAGATGATCTGCGCG
BnGTR2 LOC106405453 F FAM	BnaA02g33530D	TCTTGTTTGATTCTTCGTTTGGTTGCTAAGG
BnGTR2 LOC106405453 R	BnaA02g33530D	TGGGACTGCAGCAGTCAATA
BnGTR2 LOC106347844-LOC106424883 F HEX	BnaA06g22160D BnaC03g51560D	AACCTTCCTCCGCCGTGTAC
BnGTR2 LOC106347844-LOC106424883 R	BnaA06g22160D BnaC03g51560D	CGCCTGCTCCAACTACAAGA
BnGTR2 LOC106369007 F PET	BnaC02g42260D	GGCGTGTTCACCGTAACAGA
BnGTR2 LOC106405453 R	BnaC02g42260D	TGGGACTGCAGCAGTCAATA
BnGTR2 LOC106347844-LOC106411192 F FAM	BnaA06g22160D BnaC09g05810D	AGAGGCTGGAAAGTCATGCC

BnGTR2 LOC106347844-LOC106411192 R	BnaA06g22160D<br>BnaC09g05810D	AGAAACGCTATCTGGCCACC
BnGTR2 LOC106347844-LOC106366161 F HEX	BnaA06g22160D<br>BnaA09g06190D	AACCTTCCTCCGCCGTGTAC
BnGTR2 LOC106347844-LOC106411192 R	BnaA06g22160D<br>BnaA09g06190D	AGAAACGCTATCTGGCCACC
